# Dielectric Properties of Binary Solvent Mixtures of Dimethyl Sulfoxide with Water

**DOI:** 10.3390/ijms10031261

**Published:** 2009-03-17

**Authors:** Li-Jun Yang, Xiao-Qing Yang, Ka-Ma Huang, Guo-Zhu Jia, Hui Shang

**Affiliations:** 1 College of Electronics and Information Engineering, Sichuan University, Chengdu, 610064, P.R. China; 2 State Key Laboratory of Heavy Oil Processing, China University of Petroleum (Beijing), Changping District, Beijing 102249, P.R. China

**Keywords:** Binary Solvent, Dielectric properties, Activation energy, MD

## Abstract

In this paper, the dielectric properties of water-dimethylsulfoxide (DMSO) mixtures with different mole ratios have been investigated in the range of 1 GHz to 40 GHz at 298 K by using a molecular dynamics (MD) simulation. Only one dielectric loss peak was observed in the frequency range and the relaxation in these mixtures can be described by a single relaxation time of the Davidson-Cole. It was observed that within experimental error the dielectric relaxation can be described by the Debye-like model (*β* ≈ 1, S.M. Puranik, *et al. J. Chem. Soc. Faraday Trans.* **1992**, 88, 433 – 435). In general, the results are very consistent with the experimental measurements.

## 1. Introduction

Over the past few years, the use of microwave heating for promoting organic chemical transformations has been widely accepted by scientists [1,2]. The heating characteristics of a particular material via microwave irradiation are dependent on its dielectric properties. Therefore, the study of materials’ dielectric properties seems a crucial need [3].

Dimethylsulfoxide (DMSO) and its mixtures with other solvents (particularly water) have aroused much interest among scientists in the last decades [4–12]. The dielectric behavior of supercooled aqueous solutions of DMSO was investigated by Murthy. It was found that a 2:1 complex that was present (in DMSO solutions) in the liquid state was thermolabile and existed in an undissociated state [13]. The melting temperatures were measured by using the dielectric technique in combination with a differential scanning calorimeter. The equilibrium phase diagram of DMSO was found to be eutectic, with two compounds formed by water and DMSO at the ratios of 3:1 or 2:1 [14]. Rasmussen *et al*. studied the phase equilibrium and non-equilibrium behaviour of solutions dissolved in water and indicated that the water–DMSO system can, in all proportions, be crystallized completely, and a stable hydrate (DMSO·3H_2_O) was formed under certain conditions [15]. The energetic and structural properties of DMSO–water clusters in gas-phase by DFT and the polarizable force field have been developed by Zhang *et al*. The hydrogen bond lengths, binding energies and many-body energies of a series of DMSO–water clusters were calculated using high level *ab initio* methods [16,17].

In fact, the long-standing interest in all mixed solvents is largely due to their importance as tunable reaction media. The dielectric constant and the relaxation time, as well as the refractive indexes and other transport properties of mixed solvents, can be conveniently tuned by changing the composition [18–20]. From a molecular perspective, solvent mixtures are also interesting because they often exhibit complex structural and dynamical features, especially when the components are capable of specific interactions with one another, which may result in strong interspecies molecular associations.

A dielectric study of DMSO-water using molecular dynamics simulation (MD) is introduced in this paper. The static dielectric constant *ɛ*_0_, dielectric constant at high frequency *ɛ*_∞_, the relaxation time τ, the Cole-Cole curve and the complex permittivity spectrum have been obtained.

## 2. Interaction Potentials and Simulation Details

The mixtures with DMSO mole fractions *x_DMSO_* (*x_D_*) = 0.1, 0.21, 0.35, 0.48 and 0.91, the intermolecular potentials are described by site-site interactions given by a sum of Lennard-Jones plus Coulomb terms:

(1) $$u_{ij}(r)\;=\;4{ɛ}_{ij}\left[{\left(\sigma_{ij}/r\right)}^{12}-{\left(\sigma_{ij}/r\right)}^{6}\right]\;+\;q_{i}q_{j}/4\pi {ɛ}_{0}r$$

where *r* is the distance between sites *i* and *j* in different molecules, *q_i_* and *q_j_* are the effective charges of sites i and j, *ɛ_ij_* and *σ_ij_* are the energy and distance parameters in the Lennard-Jones potential. They are determined from the Lennard-Jones parameters of the different sites according to the “mixing” rule:

(2) $$\sigma_{ij}\;=\;\left(\sigma_{i}\;+\;\sigma_{j}\;\right)/2,\;{ɛ}_{ij}\;=\;\sqrt{{ɛ}_{i}{ɛ}_{j}}$$

where *q_i_*, *σ_i_* and *ɛ_i_* for the oxygen, sulfur, and methyl sites respectively in DMSO molecule and oxygen, hydrogen sites in water are shown in Table 1 [21].

Simulations reported in this paper were run in the NVT ensemble on systems consisting of a thousand molecules placed in a cubic box with periodic boundary conditions at an average temperature of 298 K. The box length was chosen to match the experimental density of DMSO-water mixtures solvent at 298 K. The Lennard-Jones forces were cut off at half of the box length and long-range Coulomb interactions were treated using the Ewald sum method [22]. The equations of motion were solved using combined SHAKE [23] and leap-frog [24] algorithms with a time step of 1 fs, during uninterrupted production runs lasted about 50 ps for each mixture. Then simulation were run in the NVE ensemble, all the simulations were extended up to 1.2 ns, where the first 50 ps were considered as equilibration. The starting solvent configurations for the five compositions of the mixtures have been chosen according to the specifications given in Table 2.

## 3. Results and Discussion

### 3.1. The static dielectric constant

To calculate the effective dielectric constant of a complex solute from computer simulation, it is necessary to map the properties of the solute observed in the simulation onto a simpler geometry, one amenable to analytical treatment. Specifically, the solute is approximated as a spherical cavity of volume *V* and dielectric constant *ɛ* embedded in a uniform dielectric continuum with dielectric constant *ɛ_RF_*. The charge distribution of the solute is represented as a point charge and point dipole placed at the center of the spherical cavity. From the fluctuations of the solute dipole moment, **M**, observed during a computer simulation, the absolute temperature, *T*, the volume, *V*, of the solute cavity, and the external dielectric, *ɛ_RF_*, it is then possible to determine the dielectric constant *ɛ* inside the solute cavity:

(3) $$ɛ\;=\;\frac{1+[(<M^{2}>\;-\;<M{>}^{2})/(3{ɛ}_{0}Vk_{B}T)]•2{ɛ}_{RF}/(2{ɛ}_{RF}+1)}{1-[(<M^{2}>\;-\;<M{>}^{2})/(3{ɛ}_{0}Vk_{B}T)]/(2{ɛ}_{RF}+1)}$$

where **M** is the dipole moment of the computational box, < > denotes Boltzmann ensemble averaging, *ɛ*_0_ is the dielectric constant of vacuum, *k_B_* is Boltzmann’s constant, and all of the above parameters are used SI units.

**Figure 1.**
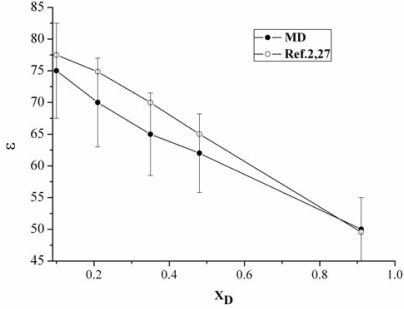
MD (full symbols) and Reference [2] and [27] (open symbols) data for the mixture’s dielectric constant vs. DMSO mole fraction.

Figure 1 describes the dielectric constant *ɛ* as a function of the composition. The error bars were estimated using the blocking method, considering that the intermolecular potentials used here lack of any polarizability effects beyond the usual dipole moment enhancement and the fact that the SPC/E underestimates water’s dielectric constant by about 10%, the MD values of *ɛ* for the mixtures are small than those from Ref. [2] and [27].

### 3.2. The relaxation time

Thermodynamic and statistical perturbation theory originally developed by Zwanzig [28] can be used to calculate relative and absolute free energies for molecularly detailed systems [29]. The change of the Gibbs free energy of a system with N particles at a given temperature, T, and pressure, P, is given by [30]:

(4) $$\Delta G\;=\;-kT\text{ln}\;<e^{-\Delta V/kT}{>}_{0}$$

Where Δ*V* is a perturbation of the Hamiltonian.

**Figure 2.**
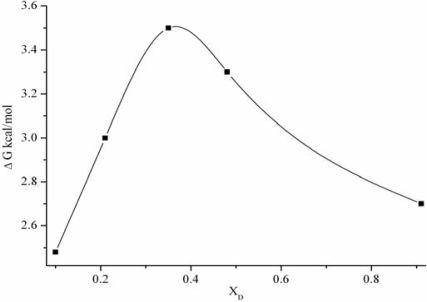
Free energy change of activation via water-DMSO solutions vs. mole fractions.

The composition dependence of the calculated values of Δ*G* is given in Figure 2. From this Figure, it can be seen that the free energy of activation increases with the concentration of the nonelectrolyte in water, reaching a maximum value of 3.5 at the concentration fraction of 0.35, whereafter it decreases. This difference can be attributed to the fact that the components are capable of specific interactions with one another.

Eyring *et al.* have applied the theory of rate processes to molecular dipole relaxation with the following equation [31]:

(5) $$\tau =\;(h/k_{B}T)\text{exp}(\Delta G/RT)$$

where Δ*G* is the free energy change of activation for the dipole relaxation, *k_B_* is Boltzmann’s constant, *h*, *R* are Planck’s constant and the molar gas constant, respectively.

**Figure 3.**
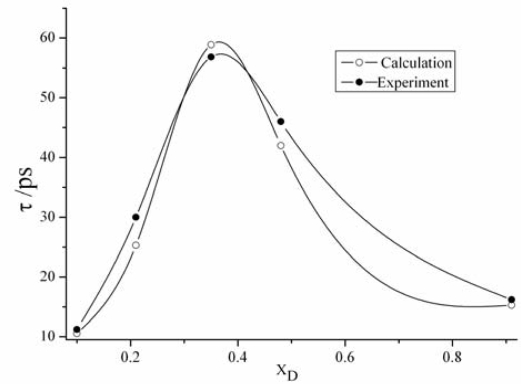
Mole fraction dependence of the relaxation time *τ* of DMSO-water mixtures at 298 K.

The mixture’s overall dielectric relaxation time *τ* is in good agreement with the experimental, except for compositions between 0.3 and 0.42, where the simulations predict significantly higher relaxation times seen from Figure 3.

In Figure 3, the open symbols show mole fraction dependence of the relaxation time is calculated by Equation 5.

### 3.3. The permittivities vs. Frequency

The complex permittivities are fitted by the nonlinear least-squares fit method to the Havriliak-Negami expression to obtain various dielectric parameters:

(6) $${ɛ}^{*}(\omega )\;=\;{ɛ}_{\infty }\;+\;({ɛ}_{0}\;+\;{ɛ}_{\infty })/{\left[1+{\left(j\omega \tau \right)}^{1-\alpha }\right]}^{\beta }$$

Where ɛ* (*ω*) is the complex permittivity at an angular frequency *ω, ɛ*_∞_ is the permittivity at high frequency, *ɛ* _∞_ is the static dielectric constant, *α* is the shape parameter representing symmetrical distribution of relaxation time and *β̣* is the shape parameter of an asymmetric relaxation curve. Equation 4 includes Cole-Cole (*β̣* =1,0 < *α* <1) [32], Davidson-Cole (*α̣ =*0, 0 < *β* < 1) [33] and Debye (*α=*0*, β̣=1*) [34] relaxation models. In our system, it is observed that under experimental error the dielectric relaxation can be described by the Debye-like (*β* ≈ 1[35]). Therefore, Equation 6 can be rewritten to Equation 7:

(7) $${ɛ}^{*}(\omega )\;=\;{ɛ}_{\infty }+({ɛ}_{0}\;+\;{ɛ}_{\infty })/(1\;+\;j\omega \tau )$$

where the complex permittivity ɛ* (*ω*) is

(8) $${ɛ}^{*}(\omega )\;=\;{ɛ}^{\prime }(\omega )\;-\;i{ɛ}^{\prime \prime }(\omega )$$

For the Debye Equation, the real *ɛ*′ and imaginary *ɛ″* contributions to the dielectric behavior can be expressed by:

(9) $$\begin{array}{l} {ɛ}^{\prime }(\omega )\;=\;{ɛ}_{\infty }\;+\;(ɛ\;-\;{ɛ}_{\infty })/(1\;+\;\omega^{2}\tau^{2})\\ {ɛ}^{\prime \prime }(\omega )\;=\;(ɛ\;-\;{ɛ}_{\infty })\omega \tau /(1\;+\;\omega^{2}\tau^{2})\\ {ɛ}_{\infty }\;\approx \;1.1n_{D}^{2} \end{array}$$

**Figure 4.**
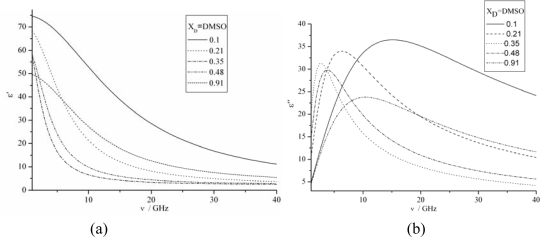
Permittivities and losses for DMSO-water mixtures at various concentrations at 298 K. (a) The real vs. frequency. (b) The imaginary vs. frequency.

Permittivity and losses for DMSO-water mixtures at 298 K are shown in Figure 4, where a single relaxation peak is observed for the entire concentration range of all the DMSO-water mixtures in the range of 1~40 GHz. Figure 4(a) obviously indicates that the higher the frequency the lower of the real *ɛ’*. Nevertheless, the real *ɛ’* decreases with the concentration of DMSO increasing, however, a completely opposite result were obtained when *x_D_* is more than 0.35. It can be addressed that the real *ɛ’* of the mixtures strongly depend on the DMSO concentration. It is expected that this phenomenon is due to the cooperative motion of DMSO-water molecules through hydrogen bonds.

**Figure 5.**
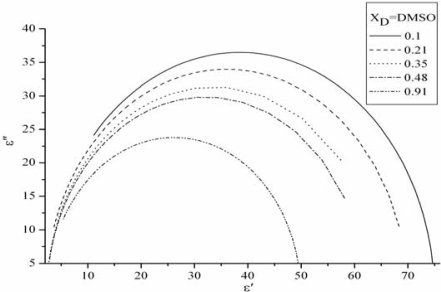
The Cole-Cole curve of DMSO-water mixture at various concentrations.

From Figure 4(b), the imaginary *ɛ″* of dielectric constant increases with the frequency till it reaches the summit, after which the imaginary part reduce gradually with the frequency. Generally, the primary process observed for various associated liquids and those water mixtures exhibit an asymmetric loss peak. In a theoretical study, it has been suggested that the motional units in the correlated domains cooperatively move and the heterogeneity of the size distribution of the domain results in the asymmetric shape of the loss peak. The concentration dependence of DMSO-water mixture solution had been calculated by Equation 9, the Cloe-Cloe diagrams can be drawn as shown in Figure 5. It can be concluded that the relation between *ɛ*″ and *ɛ″* are circle, but those centre aren’t the cross axle *ɛ*″.

## 4. Conclusions

This paper presents the picosecond dynamical profile of water-DMSO solutions over the entire range of composition. The static dielectric constant, dielectric relaxation in the frequency of microwave, as well as the microwave spectra of DMSO-water mixtures have been investigated through molecular dynamics simulation at room conditions, and this simulation has been proved to be an efficient tool for the study of molecular processes in solutions under the sufficiently wide frequency range.

## Figures and Tables

**Table 1.** L-J parameters and partial charges for water and DMSO.

	*ɛ/(kJmol^−1^)*	*σ/(nm)*	*q/(a.u.)*
O(water)	0.65	0.3165	−0.82
H(water)	0.0	0.0	0.41

O(DMSO)	0.30	0.28	−0.459
S	1.00	0.34	0.139
CH_3_	1.23	0.38	0.16

**Table 2.** Compositions of the DMSO-water mixtures. Solvent 1 is DMSO; solvent 2 is water; *x_i_* is mole fraction of solvent, *n_i_* is number of molecules of solvent i in the cubic simulation cell of edge length *L ; ρ* is the density at 298K, *n_D_* is the refractive index.

**Composition**	*x*_1_	*x*_2_	*n*_1_	*n*_2_	***L*(Å)**	*ρ*^a^**(g/cm^3^)**	**Refractive index (***n_D_***)**
1	0.1	0.9	100	900	34.05	1.0105^b^	1.3458^b^
2	0.21	0.79	210	790	36.76	1.0242^b^	1.3600^b^
3	0.35	0.65	350	650	39.0	1.0927	1.4385
4	0.48	0.52	480	520	41.38	1.0983	1.4525
5	0.91	0.09	910	90	47.93	1.0965	1.4741

^a^ Reference [25].

^b^ Reference [26].
